# Automation, organizational ambidexterity and the stability of employee relations: new tensions arising between corporate entrepreneurship, innovation management and stakeholder management

**DOI:** 10.1007/s10961-022-09987-1

**Published:** 2023-02-14

**Authors:** Martin R. W. Hiebl, David I. Pielsticker

**Affiliations:** 1 Chair of Management Accounting and Control, University of Siegen, Unteres Schloß 3, 57072 Siegen, Germany; 2 Institute of Management Control and Consulting, Johannes Kepler University Linz, Linz, Austria

**Keywords:** Automation, Corporate entrepreneurship, Organizational ambidexterity, Stakeholders, Employee relational stability, Innovation management, Tensions

## Abstract

While previous entrepreneurship research has only seldom drawn on organizational ambidexterity, the analysis of the important contemporary tensions among entrepreneurship, innovation management and strategic management issues may be facilitated by more closely analysing organizational ambidexterity in entrepreneurial settings. In this paper, we follow this thinking and more closely analyse an often applied form of corporate entrepreneurship: automation. Such automation is transferring work that was formerly conducted by humans to machines and may thus result in new tensions between corporate entrepreneurship, innovation management and the management of organizational stakeholders such as employees. The present paper investigates whether increased automation lowers the stability of firms’ relationships with their employees. In addition, we expect that this relationship is moderated by organizational ambidexterity, as employees may have perceived ambidexterity as a signal that their firm will not overly invest in exploitation only, but maintain a balance between exploitation and exploration. Drawing on stakeholder theory, previous insights into corporate entrepreneurship and a survey of German Mittelstand firms, our findings show that highly ambidextrous firms are indeed more vulnerable to automation, leading to lower employee relational stability. Our findings thus suggest that in highly ambidextrous firms, novel tensions around automation-related corporate entrepreneurship will be detrimental to the stability of the firm’s relations with one of its key stakeholder groups: employees.

## Introduction

In the past few decades, organizational ambidexterity has developed into an important and widely acknowledged research domain in management research (Li et al., 2008; O’Reilly & Tushman, 2013; Raisch & Birkinshaw, 2008). At the same time, when considering important sub-fields of management research, the ambidexterity concept has mainly been examined in strategic management and innovation management research (Cantarello et al., 2012; Guerrero, 2021). In these fields, organizational ambidexterity is generally understood as achieving a balance between the exploitation of current knowledge and the exploration of new knowledge (Andriopoulos & Lewis, 2009; March, 1991; O’Reilly & Tushman, 2013; Rojas‑Cordova et al., 2022; Rothaermel & Alexandre, 2009; Tushman and O’Reilly, 1996). By exploiting current capabilities, firms can achieve sufficient earnings, while exploration is seen as the foundation for creating new capabilities that can safeguard earnings and the firm’s further existence in the future (O’Reilly et al., 2009). So, highly ambidextrous firms manage to create products or services in an efficient way, but at the same time also constantly work on innovating or creating new products or services (Heavey et al., 2015; O’Reilly & Tushman, 2013; Raisch & Birkinshaw, 2008).

Similar concepts to organizational ambidexterity have also been investigated in entrepreneurship research. For instance, Yeganegi et al. (2019) found that employees that experience ambidexterity in their employer organization are more likely to found their own businesses afterwards. Relatedly, based on observations of the everyday behaviour of successful entrepreneurs, Volery et al. (2015) reported that these entrepreneurs show ambidexterity at the individual level. That is, they deliberately try to balance their time devoted to exploitative and explorative activities, including the identification, recognition and exploration of opportunities. All these findings suggest that for entrepreneurship to be successful, it is important to embrace a dual focus on exploitation and exploration, although such individual ambidexterity may come with severe tensions between these two modes due to entrepreneurs’ limited time and capital resources (Andriopoulos & Lewis, 2009; Volery et al., 2015). As suggested by Yeganegi et al. (2019), entrepreneurs’ ability to balance exploration and exploitation may be gained by working as an employee before founding a new business and thus experiencing organizational ambidexterity in employer organizations.

Such individual ambidexterity might not only be relevant for newly founded firms; indeed, recent research suggests that key actors’ abilities to foster ambidexterity are also relevant for corporate entrepreneurship (Burström & Wilson, 2015; Hill & Birkinshaw, 2014; Michl et al., 2013; Pan et al., 2021; Schnellbächer & Heidenreich, 2020; Weigel et al., 2022), which is generally viewed as “entrepreneurial behavior that purposefully and continuously rejuvenates the organization and shapes the scope of its operations through the recognition and exploitation of entrepreneurial opportunity” (Ireland et al., 2009, p.21). Sometimes also referred to as intrapreneurship (Leitão et al., 2020; Parker, 2011; Ramdhani et al., 2020), corporate entrepreneurship has, for example, been shown to depend on CEOs’ characteristics and their ability to drive organizational ambidexterity (Pan et al., 2021).

In summary, these clear hints on the importance of ambidexterity in existing and newly founded organizations signal that ambidexterity concepts offer a shared thread among the management, innovation and entrepreneurship literatures and may thus be a promising concept for interdisciplinary fertilization and research (Guerrero, 2021). However, while existing research on the tensions inherent to corporate entrepreneurship and ambidexterity has highlighted the relevance of individual actors for corporate renewal, newly arising tensions such as those related to technology-driven corporate entrepreneurship have not yet been explored. In this paper, we thus explore the impact of a specific case of corporate entrepreneurship that affects many contemporary organizations: automation. Generally, automation can be seen as a concept of the transfer of functions of the operational process – especially process control tasks – from humans to artificial systems, which will gradually replace human work with machine work (Arntz et al., 2017; Autor, 2015). As firms worldwide continue to strive to uphold or increase their competitiveness, they try to excel at corporate entrepreneurship and increasingly rely on automation to improve their efficiency (Jungmittag, 2021; Vanacker et al., 2021; Wright & Schultz, 2018). However, increasing investments in and managerial focus on automation may lead to tensions with innovation management. That is, an overly strong emphasis on exploitation and make it even more challenging for organizations to secure sufficient time and resources for exploration. Put differently, automation may tilt the balance between exploration and exploitation towards exploitation, which could lead to increased tensions for individuals and organizations aiming for high levels of ambidexterity and thus a balance between exploitation and exploration.

In particular, the sharp recent increase in the automation of business processes in conjunction with artificial intelligence is predicted to affect a great number of employees in industrial countries (Autor, 2015; Morrar & Arman, 2017; Vanacker et al., 2021; Wong & Ngin, 1997). Evidence suggests that benefits associated with firms’ automation, such as reducing costs, production efficiencies, and reliable production (e.g., Åström et al., 2022; Parthasarthy & Sethi, 1992), are often given greater weight than the detrimental effects of automation on employees such as lay-offs (Gasteiger & Prettner, 2017). In consequence, when pursuing corporate entrepreneurship via automation, organizations may face new tensions between efficiency gains and retaining employees. If these tensions are not resolved, employees may lose their attachment to and trust in their employers. Thus, not only from a research perspective but also for employer firms, it would be interesting to know whether automation negatively affects employee relational stability. Available research has not yet examined this question, which is why we address this theme in the present paper. In particular, we test the assumption that higher levels of automation have a negative effect on employee relational stability.

In addition, and referring back to the above-noted relevance of organizational ambidexterity as the common thread among innovation management, strategic management and entrepreneurship (Guerrero, 2021), we expect that ambidexterity plays an important role in the relationship between corporate entrepreneurship in the form of automation and employee relational stability. That is, we view automation as a form of corporate entrepreneurship (Vanacker et al., 2021) and employee relations as an important dimension of strategic stakeholder management (Freeman et al., 2010). In turn, we view the relationship between exploratory and exploitative activities as a key challenge of innovation management (Li et al., 2008; Tushman and O’Reilly, 1996). Below, we theorize that organizational ambidexterity could be the missing link to understand more fully the relationship between these instances of corporate entrepreneurship, stakeholder management and innovation management and thus address the call by Guerrero (2021) for more research investigating the role of organizational ambidexterity as the common thread among these three spheres of management. In particular, we theorize that high levels of ambidexterity create a signal to employees in the firm to retain this balance between exploitation and exploration, but if the balance is distorted due to more automation, the stability of relations with employees will suffer. (Tables 1 and 2).

We test these predictions based on survey data on German Mittelstand firms. While our results do not confirm a direct effect of automation on employee relational stability, the moderation effect involving ambidexterity receives empirical support. These findings contribute to the literature on the role of ambidexterity in corporate entrepreneurship (Burström & Wilson, 2015; Hill & Birkinshaw, 2014; Michl et al., 2013), the tensions and downsides around organizational ambidexterity (e.g., Akulava & Guerrero, 2022; Birkinshaw & Gupta, 2013; Luger et al., 2018; Montealegre et al., 2019; Rothaermel & Alexandre, 2009), to the literature on applications of stakeholder theory to phenomena of ambidexterity (e.g., Gambeta et al., 2019), and to the literature on the outcomes of automation on employee relations (Wright & Schultz, 2018).

The present study is structured as follows. In the next section, we present a short overview of the core literature relevant to our analyses and develop two formal hypotheses. Afterward, in Sect. 3, we describe our methods and discuss the descriptive characteristics of our respondents and their firms. In Sect. 4, we present the results of our analyses (see Tables 3, 4, 5 and 6), and in Sect. 5, we discuss our conclusions. In Sect. 6, we acknowledge the main limitations of this research.

## Related literature, theory and hypotheses

Corporate entrepreneurship has been highlighted as an important source of existing firms’ rejuvenation and renewal (e.g., Ireland et al., 2009; Leitão et al., 2020; Parker, 2011). As indicated above, several studies have noted that ambidexterity may help organizations realize their corporate entrepreneurship endeavours (Burström & Wilson, 2015; Hill & Birkinshaw, 2014; Michl et al., 2013; Pan et al., 2021). In addition, actors’ skills have been marked as crucial in these processes (Pan et al., 2021; Volery et al., 2015; Yeganegi et al., 2019).

However, more recently, the potential downsides of specific forms of corporate entrepreneurship have been foregrounded. For instance, Vanacker et al. (2021) recently found that corporate entrepreneurship is negatively related to firm performance in countries in which employee protection is particularly strong. They ascribed this finding to automation as a form of corporate entrepreneurship. In particular, they theorized that automation may be detrimental to employees – one of the most important stakeholder groups for many firms worldwide (e.g., Freeman et al., 2010). Vanacker et al. (2021) noted that employees and unions may thus resist automation, which could delay such corporate entrepreneurship projects or increase their cost.

While such downsides have recently been noted, the expansion of automated production processes is becoming increasingly important in many firms (e.g., Wong & Ngin, 1997). Automation can be understood as a specific form of business process innovation (Lewis et al., 2007) and thus technology-driven corporate entrepreneurship (Vanacker et al., 2021). More specifically, automation is usually considered as a concept for transferring functions of the operational process from humans to artificial systems (Autor, 2015). Automation has increased significantly in recent years and is leading to the gradual replacement of human work steps (Arntz et al., 2017; Autor, 2015). For instance, the manufacturing industry’s automation processes generally range from the use of hand tools and manual machines to the use of computer-controlled process technologies (e.g., Brownell & Merchant, 1990).

Automation, which is also referred to as the fourth industrial revolution (Morrar & Arman, 2017; Santarelli et al., 2022), offers many advantages, such as cost reduction, production efficiency, and reliable production (Parthasarthy & Sethi, 1992). In fact, to remain competitive in an increasingly globalized marketplace, firms may need to increase their efficiency by excelling at technology-driven corporate entrepreneurship endeavours such as automation (Vanacker et al., 2021; Wright & Schultz, 2018), including flexible manufacturing systems, robotics, artificial intelligence, computer-aided manufacturing, and computer-integrated manufacturing (Hayes & Jaikumar, 1988; Jungmittag, 2021; Santarelli et al., 2022).

At the same time, automation also affects many firms’ key stakeholders such as consumers, suppliers, and the wider net of stakeholders, including governments and the society (Wright & Schultz, 2018). As indicated above, the stakeholder group that may be affected most are firm employees (Autor, 2015; Morrar & Arman, 2017; Wong & Ngin, 1997). Here, automation raises new ethical, moral, but also systematic questions about how employees can keep their jobs (e.g., Parschau & Hauge, 2020; Rojas‑Cordova et al., 2022) or be included in a new collaborative form of work between humans and machines. Many employees fear losing their jobs due to the introduction of automated technologies, and this is a subject of intense recent research (e.g., Asatiani et al., 2020; Parschau & Hauge, 2020).

For a long time, such fears may not have been substantiated by evidence. That is, Bessen’s (2019) results indicate that, in particular, employment growth was initially boosted by productivity and increasing automation for more than a century, as demand was highly elastic. However, more recently, demand saturation has led to job losses, so that today’s technologies could lead to employees losing their jobs and having to make disruptive transitions to new industries in the future, which may necessitate the acquirement of new skills and occupations (Bessen, 2019). According to Gasteiger and Prettner (2017), automation can thus harm formerly trustful firm-employee relationships. From the perspective of stakeholder theory (e.g., Freeman et al., 2010; Hillman and Keim, 2001; Harrison et al., 2010), automation may be perceived by employees as the deliberate move by firms to break potentially trustful and long-lasting firm-employee relationships. Consequently, due to automation, we can expect tensions between realizing efficiency gains through corporate entrepreneurship and managing stakeholder relationships, and we expect that the stability of these employee relations is suffering due to automation.

In general, high employee relational stability is a relevant aspect for managing human resources (Barnard & Rodgers, 2000), as such stability helps to keep employee turnover and the associated costs for monitoring, adjustments, and other frictions (e.g., hiring and lay-offs) low (Failla et al., 2017; Lallemand et al., 2005). Trustful and stable employee relations and the recognition of employees are also linked to higher employee performance (Barnard & Rodgers, 2000), which is why measures attacking such employee relational stability such as automation may lower employee performance (Cropanzano et al., 2017). While firms may deliberately condone such costs arising from automation, there is also evidence that they may underestimate the detrimental employee effects associated (Carbonero et al., 2020; Makridakis, 2017; Vanacker et al., 2021). We thus propose the following hypothesis:

Hypothesis 1 (H1) An increasing degree of automation leads to a decrease in firms’ employee relational stability.

However, we do not anticipate that the relationship expressed in *H1* is universally applicable to all firms. In particular, we expect organizational ambidexterity to be an important moderator of the automation-employee relational stability relationship. As explained above, firms that feature high levels of organizational ambidexterity show a simultaneous pursuit of exploiting existing capabilities, and thus efficiency, and exploring new capabilities, thus leading to innovation and securing the long-term viability of the firm (Chandrasekaran et al., 2012; Gibson & Birkinshaw, 2004; O’Reilly & Tushman, 2013; Rojas‑Cordova et al., 2022). So as per the definition (e.g., Cao et al., 2009; Simsek, 2009), firms with high levels of organizational ambidexterity feature a balanced approach to combining high levels of efficiency gains with high levels of innovation. In addition, the existing literature has highlighted the positive relationship between organizational and individual forms of ambidexterity and corporate entrepreneurship (Burström & Wilson, 2015; Guerrero, 2021; Hill & Birkinshaw, 2014; Michl et al., 2013).

In ambidextrous firms, employees can be expected to be an important driver to reach a balance between exploration and exploitation. In fact, in certain forms of realizing organizational ambidexterity, such as contextual ambidexterity, individual employees are expected to show such balance themselves and conduct both exploration and exploitation activities (Chang, 2016; Gibson & Birkinshaw, 2004; Guerrero, 2021; Güttel & Konlechner, 2009). Not least, such individual-level ambidexterity may well equip employees to develop entrepreneurial activity (Yeganegi et al., 2019).

However, recent research has found that the pursuit of organizational ambidexterity may also come with specific tensions or outright downsides (e.g., Akulava & Guerrero, 2022; Birkinshaw & Gupta, 2013; Guerrero, 2021; Luger et al., 2018; Montealegre et al., 2019; Rothaermel & Alexandre, 2009). For instance, a strong orientation towards ambidexterity may limit a firm’s strategic opportunities as employees will expect that exploration and exploitation need to be balanced. This may be especially relevant for situations of technology-driven corporate entrepreneurship such as automation. When a firm strikes a new path by leaning more heavily towards automation, the balance between exploration and exploitation may be distorted as a higher focus on automation may lead a firm more towards exploitation (Montealegre et al., 2019), and thus away from ambidexterity. In such situations, employees may be irritated by their firms moving away from a balance between exploration and exploitation. In addition, if human tasks are increasingly transferred to robots, employees may no longer acquire knowledge on such tasks, which could in turn make their jobs less rich and limit their ability to seize opportunities that stem from knowing about both repetitive and more creative tasks (Rojas‑Cordova et al., 2022). Consequently, we expect that such employees will start to question whether the declining balance will also have an effect on themselves and whether a higher focus on automation and thus exploitation may endanger their jobs or at least reduce their jobs’ attractiveness (Rojas‑Cordova et al., 2022). Consequently, such employees may feel less attachment to their employer and thus less employee relational stability. Similar to this argument, Wright and Schultz (2018) have suggested that between employees and firms, there exist norms that are not stipulated by contract, but established by implied agreements. Wright and Schultz (2018) assume that these norms will be violated if the firm swings into a higher focus on automation. We assume that the balance between exploration and exploitation can be considered such a norm, and by implication, firms with high levels of organizational ambidexterity should feature a higher vulnerability in terms of automation-related corporate entrepreneurship, resulting in lower employee relational stability.

This notion receives support from prior research indicating that firms’ abilities to reach high levels of ambidexterity rely mainly on their employees’ ability to pursue both exploration and exploitation (e.g., Chang, 2016). So, stakeholder theory (e.g., Freeman et al., 2010) would predict that high-ambidexterity firms need to uphold close relationships with and not alienate key stakeholders such as employees to keep up their competitiveness. However, by moving more strongly towards automation, these key stakeholders may be unsettled (Rojas‑Cordova et al., 2022). This is why we expect high-ambidexterity firms which are particularly prone to automation will experience lower levels of employee relational stability.

In contrast, consider firms that are primarily focusing on efficiency gains, thus featuring a high orientation towards exploitation but focusing little on exploration and, consequently, low levels of organizational ambidexterity. In fact, we know from prior research that low levels of ambidexterity are predominantly due to higher levels of exploitation and low levels of exploration, but not vice versa (Block et al., 2013). In exploitation-oriented firms, employees may be seen more as a transactional resource and not as a source of ambidexterity. Such employees may be used to corporate entrepreneurship and new technology being implemented to improve cost efficiency further by reducing the number of employees (e.g., Merchant, 2014). In such low-ambidexterity firms, it can therefore be expected that new efficiency leaps promised by automation will not surprise employees. Thus, it will not have a big impact on employee relational stability as the firms have always sought efficiency gains and thus an exploitation orientation. All these considerations lead us to the expectation that higher levels of organizational ambidexterity are exacerbating the detrimental effect of automation on employee relational stability, as suggested in *H1*. We thus further hypothesize the following:

Hypothesis 2 (H2). The negative relationship between automation and employee relational stability (H1) is more pronounced in firms with high levels of organizational ambidexterity.

**Fig. 1:**
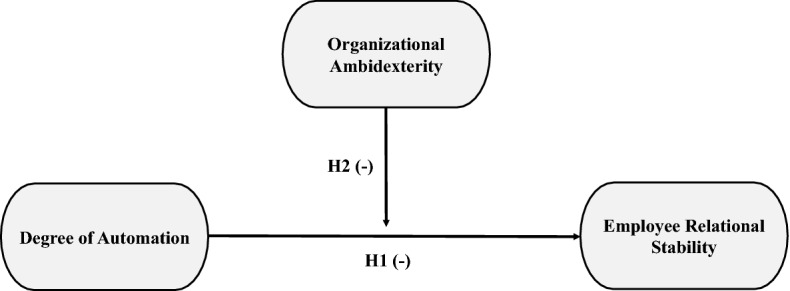
Research model

Figure 1 presents a summary visualization of our research model and the two hypotheses to be tested.

## Methods

### Sampling and data

In order to test our hypotheses, we conducted an online survey of German Mittelstand firms. Similar to previous research (e.g., Abbate et al., 2021; Dimitropoulou et al., 2021; Mitze & Makkonen, 2020), we relied on the Amadeus database to identify survey addressees. From this database, we also extracted information on the number of firm employees, firm industry affiliations, and firm contact information. Then, we manually searched for the email addresses of the top managers of each firm and specifically targeted CEOs and other members of the top management team, as Zahra (1991) has shown that these top managers usually have a broad overview of the firm’s activities. This seems especially true for Mittelstand firms, as they are usually smaller in size and thus top managers tend to have long tenures in their firms and a very close understanding of the processes going on, including aspects of automation and employee relations (Berghoff, 2006; Festing et al., 2013; Pahnke et al., 2022). In line with De Massis et al. (2018), we relied on the definition by Becker et al. (2008) and defined Mittelstand firms as those with a maximum of 3,000 employees. In addition, we focused on firms that were located close to our university since higher response rates can be expected for firms geographically proximal to a university sponsoring a survey (Bartholomew & Smith, 2006). In total, we identified a sample of 1,118 Mittelstand firms that served as our target population.

We sent out survey invitations by email to the respective firms’ top managers at the beginning of July 2020. Also, we reminded our targeted group of top managers in mid-August 2020 and assured their anonymity. A previous study by Edwards et al. (2002) has shown that incentives may positively affect response rates. Consequently, we offered our survey participants two options for incentives after they completed the survey (participants could choose to receive none, one, or both incentives):

- A donation of EUR 10 to a charity of their choice, and/or
- A detailed research report.

The majority of our questionnaire items were based on established constructs from the English language literature. We translated the respective English-language questions into German, the language used in our survey. To ensure that the questionnaire was appropriate for the survey concerning comprehensibility and structure (Hunt et al., 1982; Reynolds & Diamantopoulos, 1998), we conducted a pretest and asked ten experts (five scientists and five practitioners) for feedback on the questionnaire. Our questionnaire was translated back into the English language by a research colleague who was not involved in our research project. The aim of using the newly translated version was to check for possible translation errors (cf. Brislin, 1970). Based on the results of the pretests and the back-translation procedures, we made slight changes to the German-language questionnaire.

In total, we received 156 questionnaires that were completed in full or in part. This resulted in a response rate of approximately 14%, consistent with comparable recent studies (e.g., Abbate et al., 2021; Hossinger et al., 2021; Ng et al., 2020). The absolute response rate level may not be high when compared to meta-analytic results obtained by Baruch (1999) and Pielsticker and Hiebl (2020). However, these studies also found that response rates in management research have decreased in the last few decades, in particular for surveys addressing top managers (Cycyota & Harrison, 2006). This is why we deem the achieved response rate as satisfactory. Out of our 156 cases, we removed 26 due to a lack of information on the dependent, independent or moderator variables. Our final sample thus contains 130 cases.^1^

A further potential issue in survey studies is non-response bias (e.g., Rupp et al., 2002; van Loon, 2003). Consequently, we compared the mean values between early and late respondents for all variables involved in our study, as non-responders are considered to be similar to late respondents (Armstrong & Overton, 1977). To use the appropriate statistics for the mean value comparisons, we first tested the variables in our sample for normal distribution using a Kolmogorov–Smirnov test and a Shapiro–Wilk test, with the result that only the variables *Organizational Ambidexterity* and *Past Performance Return* were normally distributed. For these two variables, we used the t-test to examine the potential differences between early and late respondents. For the variables that did not show a normal distribution, we used the non-parametric Chi-square test (for the dichotomous variables *Retail* and *Firm Size* > *499*) or the non-parametric Mann–Whitney U-test (for all remaining variables).

As detailed in Table 1, we found no significant differences concerning the variables between early and late respondents, except for *COVID-19 Crisis Impact* and *Retail*. However, the affiliation with the *Retail* industry only shows marginally significant differences between early and late respondents and the difference regarding *COVID-19 Crisis Impact* can be explained with the timing of our survey. That is, the first respondents answered our survey in early July 2020, when the impact of the COVID-19 pandemic was more prominent in Germany and infection numbers still high. By contrast, the last respondents answered our survey in mid-August 2020, when COVID-19 infection numbers in Germany were very low (Schneble et al., 2021). Thus, we do not interpret the statistically significant difference between early and late respondents for our variable *COVID-19 Crisis Impact* as signalling non-response bias, but rather as reflecting the change in the perceived impact of the COVID-19 pandemic over the summer of 2020 (cf. Schneble et al., 2021). As we did not observe any significant differences between the core variables of interest in this study, we thus deem it unlikely that our results are affected by non-response bias.

**Table 1:** Comparison of the variables of late respondents and early respondents

Variable	Early respondents	Late respondents	*p*-value
	Mean	Mean	
Employee Relational Stability	6.19	6.31	.847
Firm Size > 499	0.53	0.42	.280
Retail	0.12	0.02	.090
Employee Loyalty	5.64	5.44	.264
Past Performance Return	4.59	4.59	1.000
Past Performance Growth	4.88	4.77	.677
COVID-19 Crisis Impact	5.00	4.02	.010
Degree of Automation	10.35	10.00	.572
Organizational Ambidexterity	167.52	167.45	.996

As found by Bowman and Ambrosini (1997), much empirical work has been published in management research that uses a single respondent approach, and respondents are frequently selected who are members of a firm’s top management team. Empirical research that opts for such a single-respondent approach is particularly confronted with potential common-method bias (Kull et al., 2018; Montabon et al., 2018). Consequently, in line with suggestions from the literature (e.g., Podsakoff et al., 2003), we have taken several established measures to avoid the development of common method bias:

- First, we guaranteed the anonymity of the respondents.
- Second, we implemented a delay between the independent and dependent variables in our questionnaire’s flow to avoid participants building their own mental models that could distort our findings.
- Third, we relied on scale items that had been pretested in prior studies and for which we additionally conducted our own pretests to ensure that our questions were simple, succinct, specific, and did not feature complicated syntax (Podsakoff et al., 2003).

Finally, to check for the potential problem of common method variance, we performed a Harman’s one-factor test (Podsakoff et al., 2003). The basic assumption of this test is that there is common method variance when only a single factor is extracted or when a factor explains most of the covariance between variables involved in a study (Podsakoff & Organ, 1986; Podsakoff et al., 2003). From our Harman’s one-factor test, the highest value for a single factor is 20.87%, which shows that no single factor explains most of the covariance between the variables involved in our study. Therefore, we have no indication that our data would suffer from common method variance.

### Measures

For constructs relying on multiple questionnaire items, we used seven-point Likert scales to measure the underlying variables. To factor-analyse these constructs, we performed principal component analyses (PCA) to determine both content and construct validity. As suggested by Field (2018), in the factor analyses, we suppressed factor loadings less than the recommended minimum 0.3. We chose the varimax rotation to maximize the loads’ dispersion within the factors (Field, 2018). For our construct and reliability analyses, we calculated Cronbach’s Alpha (should be greater than 0.7, see Field, 2018), Average Variance Extracted (AVE) (AVE value should not be less than 0.5, see Hair et al., 2019), and Composite Reliability (CR) (CR threshold should be greater than or equal to 0.7, see Hair et al., 2019). Also, the Bartlett test for item correlation (Bartlett test = 0.0) was tested. The unidimensionality was checked using Kaiser–Meyer–Olkin statistics (KMO = 0.5 as a bare minimum, see Field, 2018). Where we were able to confirm that several individual items belonged to a factor, we averaged the answers over the items of the respective construct to calculate the final values of our variables.

#### Dependent variable

*Employee Relational Stability* was measured using a scale based on the work of Johnson et al. (2004), who measured the stability of relationships with the respective firm’s suppliers. The construct was also used in studies by Yang et al. (2008) and Yang (2013). We have adapted the original questions on suppliers to fit our focus on the stability of relationships with employees. The resulting multi-item construct *Employee Relational Stability* is based on four items and is metrically scaled. All items showed sufficient reliability results (see Table 2).

#### Independent variable

Our measurement of *Degree of Automation* is based on the measurement by Inkson et al. (1970) and has been further developed by Brownell and Merchant (1990). Brownell and Merchant (1990) have used a three-part measurement construct to measure a firm’s process automation. While this measurement may seem old for an apparently recent phenomenon such as automation, we deem the contents of this construct as capable of fitting the contemporary context well. This assessment is supported by relatively recent and well-published studies that have drawn on this measurement (e.g., van Veen-Dirks, 2010). The first part of the construct requires an assessment of the degree of automation of the majority of the respondents’ firm production equipment. The evaluation is carried out on a six-level scale (for the individual wording of these six levels, see Brownell & Merchant, 1990), and using the same scale, the second part of the evaluation assesses the degree of automation of the most automated piece of equipment used in the respondent’s firm. The third part assesses the degree of automation of the final product’s quality control on a three-point scale. We have slightly adapted the third sub-question for our specific empirical setting, which involves firms from various sectors and not just manufacturing firms. That is, depending on the primary industry affiliation chosen by respondents, they were asked to assess the quality control of “their products” (for manufacturing firms), “their retail goods” (for retail firms), or “their services” (for service firms). As suggested by Brownell and Merchant (1990), the final values for our *Degree of Automation* variable were calculated by adding up the answers to the three items. That is, the higher the sum, the higher the respective firm’s level of automation.

#### Moderator variable

Our moderator variable *Organizational Ambidexterity* was measured on a 12-item construct based on the work of Lubatkin et al. (2006). The respondents were asked to indicate the degree of agreement to six statements about their firm’s exploration orientation and six statements about their firm’s exploitation orientation on a seven-point Likert scale (from “completely disagree” to “completely agree”). Based on a PCA with varimax rotation, we excluded items four and nine due to cross-loadings. The remaining items loaded on four factors, which all showed sufficient reliability results (see Table 2). The results of the factor analysis show that the exploration orientation consisted of two factors (C1 and C4), and the exploitation orientation also consisted of two factors (C2 and C3). We proceeded by computing the mean values of the two exploration factors (C1, C4) and the two exploitation factors (C2, C3).

For the following calculation of our *Organizational Ambidexterity* variable, we adopted the approach by Bedford et al. (2019). This approach is based on the notion that a high degree of *Organizational Ambidexterity* is achieved when exploitation and exploration are not only balanced but when each reaches high levels (Bedford et al., 2019). Bedford et al. (2019) propose a calculation of *Organizational Ambidexterity* by subtracting the absolute value of the difference between exploitation and exploration from seven (due to our seven-point Likert scale) and then computing the product with the exploitation and exploration scores. That is, we conceptualize the variable *Organizational Ambidexterity* as a second-order formative construct and have calculated it for a given firm *i* as follows: ORGANIZATIONAL AMBIDEXTERITY_i_ = (7—| EXPLOITATION_i_—EXPLORATION_i_ |) * EXPLOITATION_i_ * EXPLORATION_i_.

#### Controls

Based on the previous literature (e.g., Bartholomew & Smith, 2006; Wiklund & Shepherd, 2003), we introduce several control variables into our model that could affect *Employee Relational Stability*.

*Firm Size.* Smaller firms are often portrayed as offering employees more direct contact with top managers and a friendlier work environment. Consequently, employees in smaller firms have been found to show higher levels of job satisfaction (García‐Serrano, 2011; Tansel & Gazîoğlu, 2014), which may indicate that *Employee Relational Stability* is also higher in small firms. As is typical in business research (e.g., Woerter, 2012; Yu & Lee, 2017), we operationalize *Firm Size* by drawing on the number of employees. That is, we classified the firms into two size classes: the variable *Firm Size* > *499* is coded as “1” if the firm has more than 499 employees (*N* = 53), and “0” if otherwise.

*Retail.* The industry a firm operates in may influence the work environment and employees’ job satisfaction (García‐Serrano, 2011), and, by implication, *Employee Relational Stability*. In particular, the automation of retail operations is known for having special peculiarities (e.g., Begley et al., 2020). Consequently, we include the nominally scaled variable *Retail* in our analyses. This variable is coded as “1” if the firm belongs to the retail industry and “0” if otherwise.

*Employee Loyalty.* Following Loveman (1998), employee loyalty can manifest itself in service length, thus the employees’ intention to stay with the firm, which is closely related *to Employee Relational Stability*. Hence, higher *Employee Loyalty* may have a positive effect on *Employee Relational Stability*. *Employee Loyalty* was measured using a scale established by Antoncic and Antoncic (2011). The final construct was validated by a PCA, showed sufficient reliability results, and was thus calculated as the mean value of two underlying items and is metrically scaled (see Table 2). Note that the Cronbach’s *α* value for *Employee Loyalty* is low, but since this construct is only based on two items, the CR value is more meaningful for this construct (Hair et al., 2017) and indicates the construct’s sufficient validity.

*Past Performance.* An organization’s superior past performance can enable higher investments in employees’ work environment, which is closely linked to job satisfaction (Raziq & Maulabakhsh, 2015). Consequently, better-performing firms may show higher *Employee Relational Stability*. We operationalize our *Past Performance* variable by a construct suggested by Eddleston and Kellermanns (2007) that initially included eight dimensions of performance. For each of these eight dimensions, we asked our survey respondents whether their firm’s performance in the three preceding years had been “lower” or “higher” when compared with their competitors’ performance. Based on a reliability analysis, we eliminated one of the eight items. Afterward, we conducted a PCA with varimax rotation. The PCA results showed that two items related to business growth were loading on one factor, which we label as *Past Performance Growth.* The five other items were more related to profitability and loaded on a second factor, which we term *Past Performance Return*. Also, the two factors showed sufficient reliability results (see Table 2). The two *Past Performance* variables are metrically scaled and were computed as the mean value of the underlying items.

**Table 2:** Construct validity of Employee Relational Stability, Organizational Ambidexterity, Employee Loyalty, and Past Performance

Employee relational stability (reflectively measured)			
Cronbach’s *α* = .916	Composite reliability = .941	AVE = .800	Factor loadings (PCA)
The relationship between your firm and your employees is			
Unstable–stable			.888
Short-term–long-term			.908
Insecure–secure			.915
Unsteady–steady			.867

Employee loyalty (reflectively measured)			
Cronbach’s *α* = .454	Composite reliability = .794	AVE = .658	Factor loadings (PCA)
Employees talk up their organization to their friends as a great organization to work for			.811
Employees feel very little loyalty to their organization (*r*)			.811

	Factor loadings (PCA)			
Organizational Ambidexterity (Reflectively measured)	Exploitation		Exploration	
	C2	C3	C1	C4
Our firm is one that looks for novel technological ideas by thinking “outside the box.”			.870	
Our firm is one that bases its success on its ability to explore new technologies			.904	
Our firm is one that creates products or services that are innovative to the firm			.730	
Our firm is one that aggressively ventures into new market segments				.831
Our firm is one that actively targets new customer groups				.832
Our firm is one that commits to improving quality and lowering costs		.893		
Our firm is one that continuously improves the reliability of its products and services		.846		
Our firm is one that constantly surveys existing customers’ satisfaction	.793			
Our firm is one that fine-tunes what it offers to keep its current customers satisfied	.793			
Our firm is one that penetrates more deeply into its existing customer base	.730			
Cronbach’s *α*	.725	.822	.835	.686
Composite reliability (CR)	.816	.861	.875	.818
Average variance extracted (AVE)	.597	.757	.702	.691

	Factor loadings (PCA)	
Past performance (reflectively measured)	Growth	Return
How would you rate your firm’s current performance as compared with your competitors?		
Growth in sales	.915	
Growth in market shares	.914	
Growth in number of employees	.726	
Growth in profitability		.836
Return on equity		.926
Return on total assets		.932
Profit margin on sales		.885
Ability to fund growth from profits		.705
Cronbach’s *α*	.852	.932
Composite reliability (CR)	.891	.934
Average variance extracted (AVE)	.733	.741

For the variables employee relational stability and employee loyalty, one component each could be extracted from the PCA. Thus, the solution could not be rotated. So in Table 2, we display the non-rotated factor loadings.

*COVID-19 Crisis Impact*. This variable was operationalized by a single-item measure adopted from Becker et al. (2016). Becker et al. (2016) originally measured the impact of the global financial crisis in 2008. We amended their wording to fit the COVID-19 crisis and asked the participants to indicate the extent to which their firm was affected by the COVID-19 crisis on a seven-point Likert scale (from “not at all” to “very strongly”).

## Results

### Descriptive results and correlations

Table 3 shows the descriptive results of our variables. Table 4 presents a correlation matrix including the correlations between the independent variables and the dependent variable. Depending on the variables’ underlying scale levels (e.g., ordinal, metric), we have used different correlation measures (e.g., *Pearson* and *Phi*). Table 4 shows no correlation levels of 0.7 or higher and thus no indication of multicollinearity issues (Dormann et al., 2013).

All the models show sufficient predictive validity, as measured by *R*^2^. Model 3 features an *R*^2^ of 0.347. The F statistics indicate that all four models are significant at *p* < 0.01. Although our total number of observations (*N* = 130) is not large, our N would allow for up to 22 independent variables without getting into problems with statistical power (Khamis & Kepler, 2010). Since our models only feature a maximum of nine independent variables, we see no indication of problems with statistical power or overfitting.

As expected in our above discussion of control variables, the results in Model 1, which only includes the control variables (see Table 5), show that our control variable *Employee Loyalty* is positively associated with *Employee Relational Stability* (*β* = 0.550, *p* < 0.01). Model 2 shows no direct positive effect of *Degree of Automation* on *Employee Relational Stability*, which is why *H1* cannot be confirmed. In addition to the significant association between *Employee Loyalty* (*β* = 0.544, *p* < 0.01) and *Employee Relational Stability*, Model 3 suggests a significant negative effect of the interaction term (*Organizational Ambidexterity* * *Degree of Automation*) on *Employee Relational Stability* (*β* = − 0.128, *p* < 0.1), which supports *H2*.

**Fig. 2:**
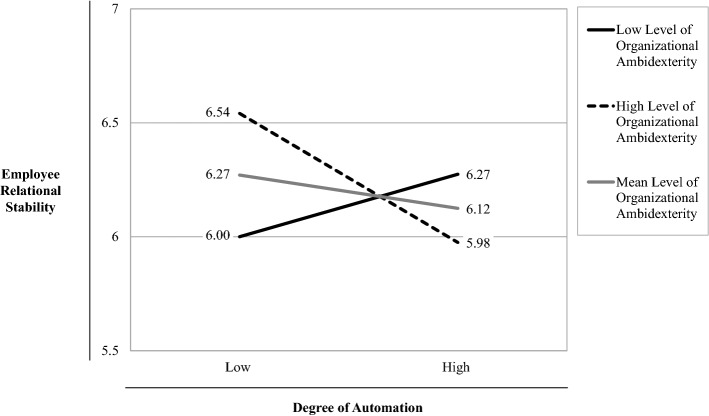
Effect of the interaction between degree of automation and organizational ambidexterity on employee relational stability

To further analyse this significant moderation effect, we conducted a simple slope analysis following Aiken and West (1991) (see Fig. 2). We computed the t-test for the simple slopes to check whether these slopes significantly differ from zero (Aiken & West, 1991; Dawson & Richter, 2006). Figure 2 shows that the solid black line representing a low level of *Organizational Ambidexterity* (mean *Organizational Ambidexterity* – 1 SD = low) has only a slightly positive but non-significant slope (*t* = 1.529, *p* > 0.1), while the dashed line representing higher levels of *Organizational Ambidexterity* (mean *Organizational Ambidexterity* + 1 SD = high) has a negative slope that significantly differs from zero (*t* = − 2.834, *p* < 0.01). These results suggest that only firms with high levels of *Organizational Ambidexterity* will we see a negative effect of *Degree of Automation* on *Employee Relational Stability*, which confirms *H2.*

### Additional analyses

Based on the hierarchical regression analysis in Table 5, Model 3 suggests a significant negative effect of the interaction term (*Organizational Ambidexterity* * *Degree of Automation*) on *Employee Relational Stability*. Since *Organizational Ambidexterity* is conceptualized as a combination of *Exploration* and *Exploitation*, we performed an additional analysis (see Table 6) to check whether the found effect of *Organizational Ambidexterity* was more due to *Exploration or Exploitation*. Again, we did not find statistically significant direct effects of either *Exploration* or *Exploitation* on *Employee Relational Stability*. However, when analysing the interaction effects, too, Model 6 in Table 6 suggests a significant negative effect of the interaction term for *Exploitation* (Exploitation* *Degree of Automation*) on *Employee Relational Stability*. For *Exploration*, we did not find such a significant interaction effect.

**Fig. 3:**
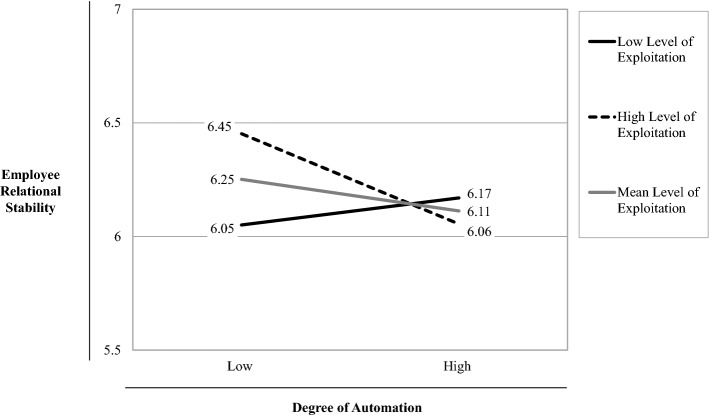
Effect of the interaction between degree of automation and exploitation on employee relational stability

Just as for our main analysis, we conducted a simple slope analysis of the effect of the interaction between *Degree of Automation* and *Exploitation* on *Employee Relational Stability* (see Fig. 3). The solid black line representing a lower level of *Exploitation* (mean *Exploitation* – 1 SD = low) has only a slightly positive but non-significant slope (*t* = 0.585, *p* > 0.1), while the dashed line representing higher levels of *Exploitation* (mean *Exploitation* + 1 SD = high) has a negative slope that significantly differs from zero (*t* = − 1.937, *p* < 0.1). These analyses show that only for firms with high levels of *Exploitation*, we found a negative effect of *Degree of Automation* on *Employee Relational Stability*, but not so for firms with high levels of *Exploration*. In summary, these additional analyses suggest that the significant effect of the interaction term between *Organizational Ambidexterity* and *Degree of Automation* on *Employee Relational Stability*, which supported *H2*, can mainly be ascribed to *Exploitation* and not to *Exploration*.

**Table 3:** Descriptives

Variables	N	Mean	Min	Max	Median	SD
Employee Relational Stability	130	6.16	2.50	7.00	6.25	.85
Firm Size > 499	130	.42	.00	1.00	.00	.49
Retail	130	.08	.00	1.00	.00	.27
Employee Loyalty	130	5.40	2.00	7.00	5.50	1.02
Past Performance Return	130	4.44	1.00	7.00	4.60	1.20
Past Performance Growth	130	4.72	1.67	7.00	4.67	1.06
COVID-19 Crisis Impact	130	4.47	1.00	7.00	5.00	1.69
Degree of Automation	130	10.09	3.00	15.00	10.00	3.48
Organizational Ambidexterity	130	164.77	30.36	343.00	163.12	60.43

**Table 4:** Correlation matrix

	Variables	N	1	2	3	4	5	6	7	8	9
1	Employee Relational Stability	130	1								
2	Firm Size > 499	130	− .022	1							
3	Retail	130	− .063	− .009	1						
4	Employee Loyalty	130	**.548**	− .012	− .015	1					
5	Past Performance Return	130	**.191**	.081	.006	**.221**	1				
6	Past Performance Growth	130	.039	**.147**	.004	**.159**	**.478**	1			
7	COVID-19 Crisis Impact	130	− .077	**.154**	.074	.086	− **.175**	.042	1		
8	Degree of Automation	130	.009	.144	− **.174**	.139	.129	**.173**	**.220**	1	
9	Organizational Ambidexterity	130	.098	.080	− .022	**.156**	**.326**	**.304**	.129	**.338**	1

Correlations significant at *p* ≤ .1 are indicated in bold; *Pearson* correlation coefficients are used for correlations between metric variables; *Point-biserial* correlation coefficients are used for correlations between metric and dichotomous variables; *Phi* values are used between dichotomous variables (for further information, see Field, 2018)

**Table 5:** Hierarchical regression analysis

Dependent variable	Employee relational stability											
	Control variables only (Model 1)				Main effect added (Model 2)				Moderation effect added (Model 3)			
Independent Variables	Stand. beta	*t* value	p value	VIF	Stand. Beta	*t* value	*p* value	VIF	Stand. beta	*t* value	*p* value	VIF
Constant		8.922	.000			8.878	.000			9.073	.000	
Firm Size > 499	.005	.061	.951	1.052	.010	.134	.893	1.061	.021	.276	.783	1.068
Retail	− .048	− .643	.521	1.007	− .060	− .781	.436	1.049	− .064	− .842	.401	1.050
Employee Loyalty	.550	7.163	.000***	1.076	.553	7.138	.000***	1.085	.544	7.058	.000***	1.091
Past Performance Return	.095	1.077	.284	1.421	.091	.996	.321	1.506	.090	.993	.323	1.506
Past Performance Growth	− .090	− 1.045	.298	1.341	− .090	− 1.033	.304	1.375	− .086	− .998	.320	1.376
COVID-19 Crisis Impact	− .101	− 1.303	.195	1.106	− .093	− 1.145	.255	1.181	− .095	− 1.190	.236	1.181
Degree of Automation					− .070	− .840	.403	1.245	− .085	− 1.027	.307	1.259
Organizational Ambidexterity					.042	.501	.617	1.290	.055	.654	.514	1.300
Degree of Automation * Organizational Ambidexterity									− .128	− 1.715	.089*	1.030
*R*^2^		.327				.331				.347		
Adjusted *R*^2^		.294				.287				.298		
*F*		9.951***				7.489***				7.090***		
*N*	130					130				130		

^*^ p < .10; ** p < .05; *** p < *.01*

**Table 6:** Hierarchical regression analysis involving separate measures for *Exploitation* and *Exploration*

Dependent Variable	Employee relational stability											
	Control variables only (Model 4)				Main effect added (Model 5)				Moderation effect added (Model 6)			
Independent Variables	Stand. beta	*t* value	*p* value	VIF	Stand. beta	*t* value	*p* value	VIF	Stand. beta	*t* value	*p* value	VIF
Constant		8.922	.000			6.590	.000			6.789	.000	
Firm Size > 499	.005	.061	.951	1.052	.017	.225	.822	1.095	.017	.217	.829	1.102
Retail	− .048	− .643	.521	1.007	− .061	− .803	.424	1.050	− 064	− .851	.396	1.050
Employee Loyalty	.550	7.163	.000***	1.076	.544	6.977	.000***	1.100	.529	6.848	.000***	1.108
Past Performance Return	.095	1.077	.284	1.421	.064	.677	.500	1.610	.089	.940	.349	1.670
Past Performance Growth	− .090	− 1.045	.298	1.341	− .084	− .957	.340	1.394	− .095	− 1.088	.279	1.430
COVID-19 Crisis Impact	− .101	− 1.303	.195	1.106	− .088	− 1.096	.275	1.173	− .065	− .804	.423	1.203
Degree of Automation					− .073	− .871	.385	1.272	− .083	− 1.002	.318	1.280
Exploitation					.087	.981	.329	1.437	.084	.958	.340	1.437
Exploration					.023	.248	.804	1.535	.010	.107	.915	1.542
Degree of Automation * Exploitation									− .158	− 1.748	.083*	1.520
Degree of Automation * Exploration									− .018	− .195	.846	1.508
*R*^2^			.327			.338				.365		
Adjusted R^2^			.294			.288				.306		
*F*			9.951***			6.796***				6.164***		
*N*			130			130				130		

^*^ p < .10; ** p < .05; *** p < *.01*

## Conclusions

The literature on organizational ambidexterity has thus far focused on strategic management and little evidence on the interplay between ambidexterity and entrepreneurship is available (Guerrero, 2021). At the same time, as suggested by Guerrero (2021), ambidexterity may be the common thread linking the management, innovation management and entrepreneurship strands of the literature that may guide research on the newly arising tensions in the contemporary business environment. In this paper, we employ this thinking to shed more light on the tensions resulting from corporate entrepreneurship linked to automation, innovation management and strategic stakeholder management. While recent research hints at the detrimental effect of automation-related corporate entrepreneurship on employees (Vanacker et al., 2021), studies directly measuring the relationship between automation and the relational stability of firms with their employees have not yet been conducted. In this paper, we address this void and additionally examine the moderating role of organizational ambidexterity that may be the missing link to better understand under which conditions increased levels of automation are detrimental to employee relational stability.

While we did not find a significant universal direct effect of automation on employee relational stability, our results indicate that for highly ambidextrous firms, higher levels of automation result in lower employee relational stability. We initially theorized that this interaction effect is due to employees in ambidextrous firms being used to a balance between exploration and exploitation, and if this balance is distorted due to a growing focus on automation and thus exploitation, the stability of employee relations will suffer. Besides our main analyses involving the combined measurement of exploration and exploitation as organizational ambidexterity, we also conducted additional analyses in which we analysed the effect of exploration and exploitation on employee relational stability separately. These latter analyses support our theory development that in ambidextrous firms that already rely heavily on exploitation, an additional focus on automation is associated with significantly lower levels of employee relational stability. Given the fact that our analyses did not reveal a direct effect of corporate entrepreneurship in the form of automation on strategic stakeholder management in terms of employee relational stability, organizational ambidexterity – as our significant moderating variable – indeed emerges from our findings as the missing link to explain the relationship between corporate entrepreneurship and strategic stakeholder management (cf. Guerrero, 2021). In particular, our findings endorse the idea that tensions around organizational ambidexterity may result in substantial effects for stakeholders (cf. Guerrero, 2021). Hence, in general, our findings reinforce Guerrero’s (2021) call to more closely examine organizational ambidexterity as the missing link between research on strategic management, innovation management and entrepreneurship.

Beyond this more general implication, our findings add to the literature in three specific ways. First, our results contribute to the so-far limited research employing organizational ambidexterity in entrepreneurship studies (Guerrero, 2021). In particular, our study focuses on corporate entrepreneurship and shows that not only are actors and their skills an important ingredient in understanding the dynamic relationship between ambidexterity and corporate entrepreneurship (Burström & Wilson, 2015; Hill & Birkinshaw, 2014; Michl et al., 2013; Pan et al., 2021; Rigtering & Behrens, 2021; Weigel et al., 2022). The new tensions arising due to technology-oriented corporate entrepreneurship such as automation may also impact this relationship. In particular, our results suggest that the potentially fragile balance between exploitation and exploration – that is, organizational ambidexterity (March, 1991; O’Reilly & Tushman, 2013) – may be increasingly tilted towards exploitation due to automation-related corporate entrepreneurship projects. In turn, employee relations may suffer, which – according to Vanacker et al. (2021) – in turn may also lower financial performance. In this way, our study may explain the archival data findings by Vanacker et al. (2021), who found that corporate entrepreneurship is detrimental to financial performance in countries with strong employee protection; Germany, the country in which we collected our empirical data, features markedly rigid employee protection regulation (Vanacker et al., 2021). In summary, as envisioned by Guerrero (2021), our study shows that organizational ambidexterity may indeed serve as a unifying thread to better understand the relationships between technology-driven corporate entrepreneurship such as automation and their joint effects on key stakeholders such as employees.

Second, our results add to the so-far limited research on the tensions around and downsides of ambidexterity (e.g., Akulava & Guerrero, 2022; Birkinshaw & Gupta, 2013; Luger et al., 2018; Montealegre et al., 2019; Rothaermel & Alexandre, 2009). The existing ambidexterity literature has overwhelmingly stressed the benefits of a firm-level balance between exploration and exploitation (e.g., Raisch & Birkinshaw, 2008; Raisch et al., 2009). Recently, it has also been shown that employees’ individual-level ambidexterity may foster their entrepreneurial activity (Yeganegi et al., 2019). Our results do not directly challenge these potential positive effects of ambidexterity since our correlation matrix also indicates a significant and positive correlation between ambidexterity and performance (see Table 4). However, in an environment of increased orientation towards corporate entrepreneurship and automation, high levels of ambidexterity may come with their idiosyncratic tensions and downsides. In particular, our results indicate that due to their ambidexterity, firms may create an implicit promise to employees that a balance between exploration and exploitation will be upheld. If, however, a firm does not uphold this balance, which can be the case with increased focus on automation and thus exploitation, employees may be irritated or disappointed, which can explain our finding on the negative impact on employee relational stability. This way, our findings also contribute to research suggesting that over extended periods of time, ambidexterity may be hard to uphold (cf. O’Reilly & Tushman, 2013).

Third, we add to the growing research on the outcomes of automation for employees. In this domain, Wright and Schultz (2018) have called for more research on the role of unwritten norms in the relationship between automation and its impact on employees. Our findings suggest that organizational ambidexterity can be considered such a norm and, if threatened through a greater reliance on automation and thus exploitation, the norm may be considered violated, which can explain why we find a negative impact of automation on employee relational stability in highly ambidextrous firms. Our findings are thus among the first to confirm empirically the predictions by Wright and Schultz (2018) on the harmful effects of automation on stakeholder relations. However, our findings qualify this effect by showing that it could only be found for highly ambidextrous firms. This suggests that Wright and Schultz’s (2018) propositions, inspired by stakeholder theory, may not hold universally and are moderated by ambidexterity. Beyond responding to the propositions by Wright and Schultz (2018), our study is generally among the first to employ thinking based on stakeholder theory to analyse ambidexterity phenomena. While Gambeta et al. (2019) recently theorized and found that good firm–employee relationships can predict organizational exploration and exploitation behaviour, we theorize and find that a firm’s level of ambidexterity may also play a role in shaping firm–employee relationships. That is, based on stakeholder theory (Freeman et al., 2010; Hillman and Keim, 2001; Harrison et al., 2010), we theorize that if implied ambidexterity norms between a firm and its stakeholders are violated, stakeholders such as employees will be irritated and their relational stability with the firm may suffer.

Our findings also hold some important implications for business practice. First, they imply that highly ambidextrous firms should examine the effects of increasing levels of automation on their employee relations extremely cautiously, while for limitedly ambidextrous firms, increasing levels of automation do not seem to be a major concern. In particular, our results suggest that firms that already feature high levels of exploitation should carefully weigh an increased focus on automation, as our findings show that the effects of increased automation in these firms are mostly detrimental to the stability of employee relations. In turn, as shown by previous research (Barnard & Rodgers, 2000; Cropanzano et al., 2017; Failla et al., 2017; Lallemand et al., 2005), such less stable relations with employees may lead to lower employee performance and higher employee turnover – two dangerous and potentially costly outcomes, which are usually not in a firm’s best interest.

## Limitations

While the discussed contributions and implications for practice are important, our underlying research, of course, is not free from limitations and leaves open important issues for future research. First, while our study adds to the so-far scant evidence on the role of ambidexterity in entrepreneurship (Guerrero, 2021), there remain many topics to be addressed in this field. Our study focused on corporate entrepreneurship, but did not address in detail how entrepreneurs – including those in newly founded firms – approach the potential tensions between increasing levels of automation in modern economies and employee relations. From the existing literature, we already know that successful entrepreneurs often possess a form of individual ambidexterity (e.g., Andriopoulos & Lewis, 2009; Volery et al., 2015; Yeganegi et al., 2019), but future research is needed on how such individual ambidexterity helps them address new tensions in their employee relations arising from technological innovation that may endanger such relations, including developments due to automation, but also related concepts such as artificial intelligence and robotics (Jungmittag, 2021; Santarelli et al., 2022; Wright & Schultz, 2018). Second, in our above theorizing, we basically assume a specific sequence of events. That is, we assume that firms are highly or limitedly ambidextrous in the first place, and then they increasingly turn towards automation (or not), which has an effect on employee relational stability. While recent literature on automation and its effects on employees (e.g., Wright & Schultz, 2018) lends support to this kind of sequence, our cross-sectional data do not allow us to test such a sequence of events directly. Studies based on longitudinal data, including in-depth case studies or time-lagged surveys, are thus needed to corroborate the theory we have developed above on the sequence of events. Third, our underlying single-respondent data may be a limitation. As we know from Podsakoff et al. (2003), respondents’ answers depend heavily on their moods, particularly relatively recent mood-building events and how they see themselves and the world around them. That is, as the respondents’ answers represent subjective assessments of their firms, these answers depend heavily on the individual respondent’s perception and, therefore, may differ from the firm’s objective situation (Podsakoff et al., 2003).

Finally, our data stem from Mittelstand firms located close to our university. The German Mittelstand is sometimes portrayed as featuring idiosyncratic benefits such as high innovativeness, but also downsides such as limited resources (Audretsch & Elston, 1997; De Massis et al., 2018; Pahnke & Welter, 2019; Pahnke et al., 2022; Weigel et al., 2022). In addition, Germany features strong employee protection regulation (Vanacker et al., 2021), which may influence employee expectations and the detrimental effect of automation on employee relational stability found for highly ambidextrous firms. Our findings thus need corroboration from other regions and types of firms.

## Appendix

See Tables

**Table 7:** Comparison of the variables of late respondents and early respondents

Variable	Early respondents	Late respondents	*p*-value
	Mean	Mean	
Exploitation	5.56	5.52	.508
Exploration	4.80	4.95	.779

^7^,

**Table 8:** Descriptives

Variables	N	Mean	Min	Max	Median	SD
Exploitation	130	5.46	2.17	7.00	5.50	0.84
Exploration	130	4.82	1.67	7.00	5.00	1.07

^8^ and

**Table 9:** Correlation matrix

	Variables	N	1	2	3	4	5	6	7	8	9	10	11
1	Employee Relational Stability	130	1										
2	Firm Size > 499	130	− .022	1									
3	Retail	130	− .063	− .009	1								
4	Employee Loyalty	130	**.548**	− .012	− .015	1							
5	Past Performance Return	130	**.191**	.081	.006	**.221**	1						
6	Past Performance Growth	130	.039	**.147**	.004	**.159**	**.478**	1					
7	COVID-19 Crisis Impact	130	− .077	**.154**	.074	.086	− **.175**	.042	1				
8	Degree of Automation	130	.009	.144	− **.174**	.139	.129	**.173**	**.220**	1			
9	Organizational Ambidexterity	130	.098	.080	− .022	**.156**	**.326**	**.304**	.129	**.338**	1		
10	Exploitation	130	**.214**	− .075	.009	**.203**	**.387**	**.155**	− .067	.122	**.617**	1	
11	Exploration	130	.103	**.160**	− .043	**.148**	**.300**	**.308**	.110	**.373**	**.921**	**.435**	1

Correlations significant at *p* ≤ .1 are indicated in bold; *Pearson* correlation coefficients are used for correlations between metric variables; *Point-biserial* correlation coefficients are used for correlations between metric and dichotomous variables; *Phi* values are used between dichotomous variables (for further information, see Field, 2018)

9

## Footnotes

1 In four of the 130 cases, information on the control variables of *Past Performance Growth* and *Employee Loyalty* was missing. Hence, we conducted Little’s missing completely at random (MCAR) test, which is frequently applied in organizational studies (e.g., Aguinis et al., 2021). The MCAR test “analyzes the pattern of missing data on all variables and compares it with the pattern expected for a random missing data process. If no significant differences are found, the missing data can be classified as MCAR” (Hair et al., 2019, p. 65). Indeed, our MCAR test did not yield significant results (p value > .1); hence, “the observed pattern [of missing data] does not differ from a random pattern” (Hair et al., 2019, p. 77). If missing data is MCAR, any usual imputation method may be chosen (Hair et al., 2019). A widespread method is mean substitution, which “replaces the missing values for a variable with the mean value of that variable calculated from all valid responses. The rationale of this approach is that the mean is the best single replacement value” (Hair et al., 2019, p. 68). We thus imputed the four missing values by using mean replacement.
